# Supplementary material for: Impact of Time-To-Surgery on the Prognosis of Patients with T1 Renal Cell Carcinoma: Implications for the COVID-19 Pandemic
Source: J Clin Med. 2022 Dec 19;11(24):7517. doi: 10.3390/jcm11247517 (PMC9786871; doi:10.3390/jcm11247517)
Supplement: Supplementary file 1 [file jcm-11-07517-s001.zip › jcm-2045378-supplementary.pdf]

Supplementary Data

Supplementary Table S1. Univariable generalized linear regression of factors influencing TTS among all patients

Variables	Coefficient	95% CI	P-values
BMI, kg/m2	-0.00	(-0.01, <0.01)	0.268
CCI	0.04	(0.01, 0.07)	0.007
PADUA score	-0.02	(-0.03, -0.01)	0.002
Sex			
Male	0		
Female	0.03	(-0.02, 0.07)	0.198
Age, years			
⩽60	0		
>60	0.04	(-0.01, 0.08)	0.134
Maximal tumor diameter at presentation, cm			
⩽4	0		
>4	-0.08	(-0.12, -0.04)	<0.001
ASA score			
I	0		
II	0.01	(-0.04, 0.06)	0.638
III	0.06	(-0.02, 0.14)	0.145
WBC, x10^9/L			
⩽10	0		
>10	-0.04	(-0.13, 0.04)	0.330
Hb, g/L			
⩽110	0		
>110	0.00	(-0.08, 0.09)	0.949
PLT, x10^9/L			
⩽100	0		
>100	0.06	(-0.28, 0.40)	0.724
Serum creatine, µmol/L			
⩽115	0		
>115	-0.01	(-0.11, 0.09)	0.857
Serum calcium, mmol/L			
<2.25	0		
⩾2.25	-0.01	(-0.06, 0.03)	0.515
ALP, U/L			
⩽110	0		
>110	0.02	(-0.09, 0.13)	0.738
Preoperative maximal tumor diameter, cm			
⩽4	0		

>4	-0.04	(-0.08, <0.01)	0.053

*Statistically significant at alpha = 0.1.

Abbreviations: TTS, time-to-surgery; CI, confidence interval; BMI, body mass index; CCI, Charlson comorbidity index; PADUA, Preoperative Aspects and Dimensions Used for an Anatomical; ASA, American Society of Anesthesiologists; WBC, white blood cell; Hb, hemoglobin; PLT, platelet; ALP, alkaline phosphatase; UISS, University of California Los Angeles Integrated Staging System.

Supplementary Table S2. Clinical characteristics of T1a patients stratified by TTS

	Study cohort (N=329)	TTS≤ 5 weeks (N=256)	TTS> 5 weeks (N=73)	P-values
BMI, kg/m^2, median (IQR)	23.9 (21.8, 25.7)	23.9 (21.9, 25.7)	23.9 (21.8, 25.6)	0.806
CCI, median (IQR)	2.0 (2.0, 2.0)	2.0 (2.0, 2.0)	2.0 (2.0, 2.0)	0.818
Age, years, n (%)				0.655
≤60	241 (73.3)	189 (73.8)	52 (71.2)	
>60	88 (26.7)	67 (26.2)	21 (28.8)	
Sex, n (%)				0.675
Male	216 (65.7)	166 (64.8)	50 (68.5)	
Female	113 (34.3)	90 (35.2)	23 (31.5)	
ASA score, n (%)				0.977
I	121 (36.8)	95 (37.1)	26 (35.6)	
II	184 (55.9)	142 (55.5)	42 (57.5)	
III	24 (7.3)	19 (7.4)	5 (6.8)	
Preoperative maximal tumor diameter, cm, n (%)				0.195
≤4	294 (89.4)	232 (90.6)	62 (84.9)	
>4	35 (10.6)	24 (9.4)	11 (15.1)	
WBC, x10^9/L, n (%)				1.000
<=10	313 (95.1)	243 (94.9)	70 (95.9)	
>10	16 (4.9)	13 (5.1)	3 (4.1)	
Hb, g/L, n (%)				0.587
≤110	21 (6.4)	18 (7.0)	3 (4.1)	
>110	308 (93.6)	238 (93.0)	70 (95.9)	
PLT, x10^9/L, n (%)				1.000
≤100	2 (0.6)	2 (0.8)	0 (0.0)	
>100	327 (99.4)	254 (99.2)	73 (100.0)	
Serum creatine, µmol/L, n (%)				0.495
≤115	316 (96.0)	247 (96.5)	69 (94.5)	
>115	13 (4.0)	9 (3.5)	4 (5.5)	
Serum calcium, mmol/L, n (%)				1.000
<2.25	136 (41.3)	106 (41.4)	30 (41.1)	
≥2.25	193 (58.7)	150 (58.6)	43 (58.9)	
ALP, U/L, n (%)				0.713
≤110	318 (96.7)	248 (96.9)	70 (95.9)	
>110	11 (3.3)	8 (3.1)	3 (4.1)	
PADUA score, median (IQR)	9.0 (8.0, 10.0)	9.0 (8.0, 10.0)	9.0 (8.0, 9.0)	0.582
Necrosis in pathological tumor tissue, n (%)				0.741

No	316 (96.0)	245 (95.7)	71 (97.3)	
Yes	13 (4.0)	11 (4.3)	2 (2.7)	
UISS grade, n (%)				0.793
Low-risk	177 (53.8)	137 (53.5)	40 (54.8)	
Intermediate-risk	117 (35.6)	90 (35.2)	27 (37.0)	
High-risk	35 (10.6)	29 (11.3)	6 (8.2)	
Surgical approach, n (%)				0.394
Open	106 (32.2)	86 (33.6)	20 (27.4)	
Laparoscopic	223 (67.8)	170 (66.4)	53 (72.6)	
Surgical resection method, n (%)				1.000
Partial nephrectomy	225 (68.4)	175 (68.4)	50 (68.5)	
Radical nephrectomy	104 (31.6)	81 (31.6)	23 (31.5)	
Operation time, min, n (%)				0.082
≤150	188 (57.1)	153 (59.8)	35 (47.9)	
>150	141 (42.9)	103 (40.2)	38 (52.1)	
Intraoperative bleeding, ml, n (%)				0.892
≤50	203 (61.7)	157 (61.3)	46 (63.0)	
>50	126 (38.3)	99 (38.7)	27 (37.0)	
Surgical complication grade, n (%)				0.660
None	250 (76.0)	194 (75.8)	56 (76.7)	
I	68 (20.7)	52 (20.3)	16 (21.9)	
II-IV	11 (3.3)	10 (3.9)	1 (1.4)	
Surgical wound infection, n (%)				1.000
No	319 (97.0)	248 (96.9)	71 (97.3)	
Yes	10 (3.0)	8 (3.1)	2 (2.7)	
Postoperative blood transfusion, n (%)				0.590
No	324 (98.5)	251 (98.0)	73 (100.0)	
Yes	5 (1.5)	5 (2.0)	0 (0.0)	

*Statistically significant at alpha = 0.05.

Abbreviations: TTS, time-to-surgery; BMI, body mass index; IQR, interquartile range; CCI, Charlson comorbidity index; ASA, American Society of Anesthesiologists; WBC, white blood cell; Hb, hemoglobin; PLT, platelet; ALP, alkaline phosphatase; PADUA, Preoperative Aspects and Dimensions Used for an Anatomical; UISS, University of California Los Angeles Integrated Staging System.

Supplementary Table S3. Univariable Cox regression for DFS and OS among T1a patients

	DFS			OS		
Variables	HR	95% CI	P-values	HR	95% CI	P-values
TTS, weeks						
≤5	1.00			1.00		
>5	1.96	(0.68, 5.65)	0.212	1.22	(0.26, 5.78)	0.806
BMI, kg/m2	0.98	(0.86, 1.13)	0.830	1.09	(0.95, 1.26)	0.205
CCI	1.11	(0.59, 2.10)	0.736	1.45	(0.80, 2.64)	0.221
PADUA score	1.05	(0.78, 1.42)	0.757	1.13	(0.76, 1.67)	0.542
Sex						
Male	1.00			1.00		
Female	0.39	(0.11, 1.37)	0.143	0.21	(0.03, 1.69)	0.143
Age, years						
≤60	1.00			1.00		
>60	4.17	(1.58, 10.96)	0.004	11.50	(2.44, 54.21)	0.002
ASA score						
I	1.00			1.00		
II	1.07	(0.33, 3.50)	0.912	0.46	(0.09, 2.28)	0.339
III	5.87	(1.44, 23.97)	0.014	9.46	(1.91, 46.95)	0.006
WBC, x10^9/L						
≤10	1.00			1.00		
>10	1.43	(0.19, 10.76)	0.731	NA		
Hb, g/L						
≤110	1.00			1.00		
>110	0.28	(0.08, 0.98)	0.047	0.50	(0.06, 4.05)	0.519
PLT, x10^9/L						
≤100	1.00			1.00		
>100	NA			NA		
Serum creatine, µmol/L						
≤115	1.00			1.00		
>115	NA			NA		
Serum calcium, mmol/L						
<2.25	1.00			1.00		
≥2.25	0.59	(0.23, 1.53)	0.279	0.83	(0.23, 2.95)	0.771
ALP, U/L						
≤110	1.00			1.00		
>110	NA			NA		
Preoperative maximal tumor diameter, cm						

≤4	1.00			1.00		
>4	2.41	(0.79, 7.40)	0.124	1.94	(0.41, 9.15)	0.403
Surgical approach						
Open	1.00			1.00		
Laparoscopic	0.72	(0.28, 1.88)	0.504	0.96	(0.27, 3.41)	0.945
Surgical resection method						
Partial nephrectomy	1.00			1.00		
Radical nephrectomy	1.44	(0.55, 3.75)	0.458	0.65	(0.17, 2.56)	0.540
Operation time, min						
≤150	1.00			1.00		
>150	1.58	(0.61, 4.11)	0.345	1.41	(0.41, 4.89)	0.585
Intraoperative bleeding, ml						
≤50	1.00			1.00		
>50	1.06	(0.40, 2.78)	0.907	1.01	(0.28, 3.57)	0.992
Surgical complication grade						
None	1.00			1.00		
I	4.16	(1.50, 11.49)	0.006	1.67	(0.33, 8.37)	0.531
II-IV	4.55	(0.55, 37.40)	0.159	6.01	(0.68, 53.35)	0.107
Surgical wound infection						
No	1.00			1.00		
Yes	3.39	(0.44, 26.00)	0.241	6.68	(0.81, 54.87)	0.077
Postoperative blood transfusion						
No	1.00			1.00		
Yes	NA			NA		
Necrosis in pathological tumor tissue						
No	1.00			1.00		
Yes	3.53	(0.80, 15.49)	0.095	2.84	(0.36, 22.51)	0.324
UISS grade						
Low-risk	1.00			1.00		
Intermediate-risk	8.53	(1.91, 38.19)	0.005	10.58	(1.32, 84.87)	0.026
High-risk	7.05	(1.17, 42.62)	0.033	4.24	(0.26, 68.76)	0.309

*Statistically significant at alpha = 0.05.

Abbreviations: DFS, disease-free survival; OS overall survival; HR, hazard ratio; CI, confidence interval; TTS, time-to-surgery; BMI, body mass index; CCI, Charlson comorbidity index; PADUA, Preoperative Aspects and Dimensions Used for an Anatomical; ASA, American Society of Anesthesiologists; WBC, white blood cell; Hb, hemoglobin; PLT, platelet; ALP, alkaline phosphatase; UISS, University of California Los Angeles Integrated Staging System.

Supplementary Table S4. Clinical characteristics of T1a patients stratified by TTS and adjusted by IPTW

	TTS≤ 5 weeks (N=328.6)	TTS> 5 weeks (N=327.4)	P-values	SMD
BMI, kg/m^2, median (IQR)	23.88 (21.78, 25.62)	23.74 (21.49, 25.61)	0.825	0.074
CCI, median (IQR)	2.00 (2.00, 2.00)	2.00 (2.00, 2.00)	0.818	0.007
Age, years, n (%)			0.816	0.032
≤60	241.7 (73.5)	245.4 (75.0)		
>60	86.9 (26.5)	82.0 (25.0)		
Sex, n (%)			0.937	0.012
Male	215.8 (65.7)	216.8 (66.2)		
Female	112.8 (34.3)	110.6 (33.8)		
ASA score, n (%)			0.943	0.049
I	121.3 (36.9)	126.5 (38.6)		
II	182.9 (55.7)	174.4 (53.3)		
III	24.3 (7.4)	26.5 (8.1)		
Preoperative maximal tumor diameter, cm, n (%)			0.974	0.004
≤4	294.8 (89.7)	293.3 (89.6)		
>4	33.8 (10.3)	34.1 (10.4)		
WBC, x10^9/L, n (%)			0.883	0.021
<=10	312.6 (95.1)	312.9 (95.6)		
>10	16.0 (4.9)	14.5 (4.4)		
Hb, g/L, n (%)			0.861	0.03
≤110	21.2 (6.4)	23.6 (7.2)		
>110	307.5 (93.6)	303.8 (92.8)		
PLT, x10^9/L, n (%)			0.161	0.121
≤100	2.4 (0.7)	0.0 (0.0)		
>100	326.2 (99.3)	327.4 (100.0)		
Serum creatine, μmol/L, n (%)			0.786	0.031
≤115	315.9 (96.1)	316.6 (96.7)		
>115	12.8 (3.9)	10.8 (3.3)		
Serum calcium, mmol/L, n (%)			0.86	0.025
<2.25	136.0 (41.4)	139.6 (42.6)		
≥2.25	192.6 (58.6)	187.8 (57.4)		
ALP, U/L, n (%)			0.936	0.01
≤110	317.7 (96.7)	317.1 (96.9)		
>110	10.9 (3.3)	10.3 (3.1)		
PADUA score, median (IQR)	9.00 (8.00, 10.00)	9.00 (8.00, 9.00)	0.93	0.043
Necrosis in pathological tumor tissue, n (%)			0.556	0.101

No	315.2 (95.9)	306.7 (93.7)		
Yes	13.4 (4.1)	20.7 (6.3)		
UISS grade, n (%)			0.774	0.102
Low-risk	178.7 (54.4)	184.9 (56.5)		
Intermediate-risk	115.5 (35.1)	117.6 (35.9)		
High-risk	34.5 (10.5)	24.8 (7.6)		
Surgical approach, n (%)			0.926	0.014
Open	106.1 (32.3)	103.6 (31.6)		
Laparoscopic	222.6 (67.7)	223.8 (68.4)		
Surgical resection method, n (%)			0.892	0.02
Partial nephrectomy	224.9 (68.4)	221.0 (67.5)		
Radical nephrectomy	103.7 (31.6)	106.4 (32.5)		
Operation time, min, n (%)			0.764	0.042
≤150	188.5 (57.4)	194.5 (59.4)		
>150	140.1 (42.6)	132.9 (40.6)		
Intraoperative bleeding, ml, n (%)			0.91	0.016
≤50	202.0 (61.5)	203.8 (62.2)		
>50	126.7 (38.5)	123.6 (37.8)		
Surgical complication grade, n (%)			0.952	0.047
None	249.3 (75.9)	246.3 (75.2)		
I	68.4 (20.8)	72.3 (22.1)		
II-IV	11.0 (3.3)	8.8 (2.7)		
Surgical wound infection, n (%)			0.917	0.015
No	318.5 (96.9)	316.4 (96.6)		
Yes	10.2 (3.1)	11.0 (3.4)		
Postoperative blood transfusion, n (%)			0.027	0.186
No	323.0 (98.3)	327.4 (100.0)		
Yes	5.6 (1.7)	0.0 (0.0)		

*Statistically significant at alpha = 0.05.

Abbreviations: TTS, time-to-surgery; IPTW, inverse probability-treatment weighting; BMI, body mass index; IQR, interquartile range; SMD, standardized mean difference; CCI, Charlson comorbidity index; ASA, American Society of Anesthesiologists; WBC, white blood cell; Hb, hemoglobin; PLT, platelet; ALP, alkaline phosphatase; PADUA, Preoperative Aspects and Dimensions Used for an Anatomical; UISS, University of California Los Angeles Integrated Staging System.

Supplementary Table S5. Clinical characteristics of T1b patients stratified by TTS

	Study cohort (N=331)	TTS≤ 5 weeks (N=283)	TTS> 5 weeks (N=48)	P-values
BMI, kg/m^2, median (IQR)	23.5 (21.4, 25.3)	23.4 (21.5, 25.3)	23.5 (21.1, 25.1)	0.646
CCI, median (IQR)	2.0 (2.0, 2.0)	2.0 (2.0, 2.0)	2.0 (2.0, 2.0)	0.296
Age, years, n (%)				0.728
≤60	241 (72.8)	207 (73.1)	34 (70.8)	
>60	90 (27.2)	76 (26.9)	14 (29.2)	
Sex, n (%)				0.251
Male	218 (65.9)	190 (67.1)	28 (58.3)	
Female	113 (34.1)	93 (32.9)	20 (41.7)	
ASA score, n (%)				0.394
I	98 (29.6)	87 (30.7)	11 (22.9)	
II	198 (59.8)	168 (59.4)	30 (62.5)	
III	35 (10.6)	28 (9.9)	7 (14.6)	
Preoperative maximal tumor diameter, cm, n (%)				0.497
≤4	45 (13.6)	37 (13.1)	8 (16.7)	
>4	286 (86.4)	246 (86.9)	40 (83.3)	
WBC, x10^9/L, n (%)				0.750
<=10	311 (94.0)	265 (93.6)	46 (95.8)	
>10	20 (6.0)	18 (6.4)	2 (4.2)	
Hb, g/L, n (%)				0.763
≤110	24 (7.3)	20 (7.1)	4 (8.3)	
>110	307 (92.7)	263 (92.9)	44 (91.7)	
PLT, x10^9/L, n (%)				0.145
≤100	1 (0.3)	0 (0.0)	1 (2.1)	
>100	330 (99.7)	283 (100.0)	47 (97.9)	
Serum creatine, µmol/L, n (%)				1.000
≤115	311 (94.0)	266 (94.0)	45 (93.8)	
>115	20 (6.0)	17 (6.0)	3 (6.3)	
Serum calcium, mmol/L, n (%)				0.752
<2.25	131 (39.6)	111 (39.2)	20 (41.7)	
≥2.25	200 (60.4)	172 (60.8)	28 (58.3)	
ALP, U/L, n (%)				0.437
≤110	317 (95.8)	272 (96.1)	45 (93.8)	
>110	14 (4.2)	11 (3.9)	3 (6.3)	

PADUA score, median (IQR)	10.0 (8.0, 11.0)	10.0 (9.0, 11.0)	9.0 (8.0, 11.0)	0.113
Necrosis in pathological tumor tissue, n (%)				1.000
No	302 (91.2)	258 (91.2)	44 (91.7)	
Yes	29 (8.8)	25 (8.8)	4 (8.3)	
UISS grade, n (%)				0.463
Low-risk	166 (50.2)	145 (51.2)	21 (43.8)	
Intermediate-risk	118 (35.6)	97 (34.3)	21 (43.8)	
High-risk	47 (14.2)	41 (14.5)	6 (12.5)	
Surgical approach, n (%)				0.620
Open	109 (32.9)	95 (33.6)	14 (29.2)	
Laparoscopic	222 (67.1)	188 (66.4)	34 (70.8)	
Surgical resection method, n (%)				0.842
Partial nephrectomy	61 (18.4)	53 (18.7)	8 (16.7)	
Radical nephrectomy	270 (81.6)	230 (81.3)	40 (83.3)	
Operation time, min, n (%)				0.639
≤150	153 (46.2)	129 (45.6)	24 (50.0)	
>150	178 (53.8)	154 (54.4)	24 (50.0)	
Intraoperative bleeding, ml, n (%)				0.753
≤50	191 (57.7)	162 (57.2)	29 (60.4)	
>50	140 (42.3)	121 (42.8)	19 (39.6)	
Surgical complication grade, n (%)				0.769
None	273 (82.5)	234 (82.7)	39 (81.3)	
I	49 (14.8)	42 (14.8)	7 (14.6)	
II-IV	9 (2.7)	7 (2.5)	2 (4.2)	
Surgical wound infection, n (%)				1.000
No	325 (98.2)	278 (98.2)	47 (97.9)	
Yes	6 (1.8)	5 (1.8)	1 (2.1)	
Postoperative blood transfusion, n (%)				1.000
No	324 (97.9)	277 (97.9)	47 (97.9)	
Yes	7 (2.1)	6 (2.1)	1 (2.1)	

*Statistically significant at alpha = 0.05.

Abbreviations: TTS, time-to-surgery; BMI, body mass index; IQR, interquartile range; CCI, Charlson comorbidity index; ASA, American Society of Anesthesiologists; WBC, white blood cell; Hb, hemoglobin; PLT, platelet; ALP, alkaline phosphatase; PADUA, Preoperative Aspects and Dimensions Used for an Anatomical; UISS, University of

California Los Angeles Integrated Staging System.

Supplementary Table S6. Univariable Cox regression for DFS and OS among T1b patients

	DFS			OS		
Variables	HR	95% CI	P-values	HR	95% CI	P-values
TTS, weeks						
≤5	1.00			1.00		
>5	2.70	(1.38, 5.28)	0.004	2.27	(1.02, 5.05)	0.044
BMI, kg/m2	1.03	(0.95, 1.13)	0.464	1.08	(0.98, 1.18)	0.127
CCI	1.33	(1.10, 1.60)	0.003	1.43	(1.19, 1.71)	<0.001
PADUA score	1.16	(0.97, 1.38)	0.097	1.04	(0.85, 1.26)	0.717
Sex						
Male	1.00			1.00		
Female	0.68	(0.35, 1.31)	0.249	0.39	(0.16, 0.93)	0.034
Age, years						
≤60	1.00			1.00		
>60	2.69	(1.48, 4.87)	0.001	2.56	(1.30, 5.04)	0.007
ASA score						
I	1.00			1.00		
II	1.26	(0.59, 2.69)	0.554	1.78	(0.67, 4.73)	0.247
III	2.75	(1.09, 6.95)	0.032	4.12	(1.34, 12.61)	0.013
WBC, x10^9/L						
≤10	1.00			1.00		
>10	2.52	(0.99, 6.40)	0.052	2.71	(0.95, 7.69)	0.061
Hb, g/L						
≤110	1.00			1.00		
>110	0.20	(0.10, 0.40)	<0.001	0.28	(0.11, 0.67)	0.004
PLT, x10^9/L						
≤100	1.00			1.00		
>100	0.00	(0.00, 0.05)	<0.001	0.12	(0.02, 0.88)	0.037
Serum creatine, µmol/L						
≤115	1.00			1.00		
>115	2.28	(0.90, 5.79)	0.083	3.25	(1.26, 8.41)	0.015
Serum calcium, mmol/L						
<2.25	1.00			1.00		
≥2.25	0.90	(0.49, 1.64)	0.729	0.97	(0.48, 1.93)	0.922
ALP, U/L						
≤110	1.00			1.00		
>110	2.56	(0.91, 7.19)	0.074	1.71	(0.41, 7.15)	0.463
Preoperative maximal						

tumor diameter, cm						
≤4	1.00			1.00		
>4	1.12	(0.44, 2.85)	0.805	1.46	(0.45, 4.79)	0.529
Surgical approach						
Open	1.00			1.00		
Laparoscopic	0.60	(0.33, 1.09)	0.094	0.51	(0.26, 1.01)	0.052
Surgical resection method						
Partial nephrectomy	1.00			1.00		
Radical nephrectomy	1.58	(0.62, 4.00)	0.339	1.46	(0.51, 4.14)	0.478
Operation time, min						
≤150	1.00			1.00		
>150	1.95	(1.03, 3.68)	0.039	2.22	(1.06, 4.65)	0.034
Intraoperative bleeding, ml						
≤50	1.00			1.00		
>50	1.99	(1.09, 3.64)	0.025	2.47	(1.22, 4.99)	0.012
Surgical complication grade						
None	1.00			1.00		
I	2.35	(1.21, 4.54)	0.011	2.74	(1.31, 5.73)	0.007
II-IV	4.49	(1.37, 14.80)	0.013	6.51	(1.93, 21.96)	0.003
Surgical wound infection						
No	1.00			1.00		
Yes	4.97	(1.53, 16.14)	0.008	6.69	(2.03, 22.03)	0.002
Postoperative blood transfusion						
No	1.00			1.00		
Yes	6.35	(1.96, 20.54)	0.002	8.86	(2.70, 29.13)	<0.001
Necrosis in pathological tumor tissue						
No	1.00			1.00		
Yes	3.35	(1.55, 7.23)	0.002	2.76	(1.07, 7.16)	0.037
UISS grade						
Low-risk	1.00			1.00		
Intermediate-risk	3.42	(1.55, 7.57)	0.002	4.28	(1.67, 10.94)	0.002
High-risk	7.04	(3.11, 15.94)	<0.001	7.68	(2.88, 20.47)	<0.001

*Statistically significant at alpha = 0.05.

Abbreviations: DFS, disease-free survival; OS overall survival; HR, hazard ratio; CI, confidence interval; TTS, time-to-surgery; BMI, body mass index; CCI, Charlson comorbidity index; PADUA, Preoperative Aspects and Dimensions Used for an Anatomical; ASA, American Society of Anesthesiologists; WBC, white blood cell; Hb, hemoglobin; PLT, platelet; ALP, alkaline phosphatase; UISS, University of California Los Angeles Integrated Staging System.

Supplementary Table S7. Clinical characteristics of T1b patients stratified by TTS and adjusted by IPTW

	TTS≤ 5 weeks (N=328.6)	TTS> 5 weeks (N=327.4)	P-values	SMD
BMI, kg/m^2, median (IQR)	23.40 (21.47, 25.30)	23.46 (20.75, 24.90)	0.571	0.012
CCI, median (IQR)	2.00 (2.00, 2.00)	2.00 (2.00, 2.00)	0.385	0.055
Age, years, n (%)			0.808	0.041
≤60	239.5 (72.6)	233.8 (70.8)		
>60	90.2 (27.4)	96.5 (29.2)		
Sex, n (%)			0.96	0.008
Male	216.7 (65.7)	215.8 (65.3)		
Female	113.0 (34.3)	114.5 (34.7)		
ASA score, n (%)			0.87	0.088
I	98.0 (29.7)	104.8 (31.7)		
II	197.4 (59.9)	184.5 (55.8)		
III	34.2 (10.4)	41.0 (12.4)		
Preoperative maximal tumor diameter, cm, n (%)			0.858	0.029
≤4	45.1 (13.7)	48.5 (14.7)		
>4	284.6 (86.3)	281.8 (85.3)		
WBC, x10^9/L, n (%)			0.577	0.092
<=10	309.8 (94.0)	317.0 (96.0)		
>10	19.9 (6.0)	13.3 (4.0)		
Hb, g/L, n (%)			0.718	0.055
≤110	22.9 (6.9)	18.5 (5.6)		
>110	306.8 (93.1)	311.8 (94.4)		
PLT, x10^9/L, n (%)			0.33	0.078
≤100	0.0 (0.0)	1.0 (0.3)		
>100	329.7 (100.0)	329.3 (99.7)		
Serum creatine, μmol/L, n (%)			0.838	0.035
≤115	309.7 (93.9)	307.4 (93.1)		
>115	20.0 (6.1)	22.9 (6.9)		
Serum calcium, mmol/L, n (%)			0.946	0.011
<2.25	131.0 (39.7)	129.4 (39.2)		
≥2.25	198.7 (60.3)	200.9 (60.8)		
ALP, U/L, n (%)			0.851	0.028
≤110	315.5 (95.7)	314.1 (95.1)		
>110	14.2 (4.3)	16.2 (4.9)		
PADUA score, median (IQR)	10.00 (8.00, 11.00)	9.00 (8.00, 11.00)	0.911	0.029
Necrosis in pathological			0.973	0.006

tumor tissue, n (%)				
No	300.6 (91.2)	300.6 (91.0)		
Yes	29.1 (8.8)	29.7 (9.0)		
UISS grade, n (%)			0.884	0.082
Low-risk	165.7 (50.3)	167.0 (50.6)		
Intermediate-risk	117.2 (35.5)	125.0 (37.8)		
High-risk	46.8 (14.2)	38.3 (11.6)		
Surgical approach, n (%)			0.952	0.01
Open	107.7 (32.7)	106.3 (32.2)		
Laparoscopic	222.0 (67.3)	224.0 (67.8)		
Surgical resection method, n (%)			0.76	0.051
Partial nephrectomy	61.1 (18.5)	60.8 (18.4)		
Radical nephrectomy	268.6 (81.5)	269.5 (81.6)		
Operation time, min, n (%)			0.76	0.051
≤150	151.3 (45.9)	143.2 (43.4)		
>150	178.4 (54.1)	187.1 (56.6)		
Intraoperative bleeding, ml, n (%)			0.921	0.017
≤50	190.9 (57.9)	194.0 (58.7)		
>50	138.8 (42.1)	136.3 (41.3)		
Surgical complication grade, n (%)			0.926	0.062
None	272.6 (82.7)	280.0 (84.8)		
I	48.5 (14.7)	41.6 (12.6)		
II-IV	8.6 (2.6)	8.8 (2.7)		
Surgical wound infection, n (%)			0.926	0.014
No	323.6 (98.2)	323.6 (98.0)		
Yes	6.0 (1.8)	6.7 (2.0)		
Postoperative blood transfusion, n (%)			0.983	0.003
No	322.7 (97.9)	323.1 (97.8)		
Yes	7.0 (2.1)	7.2 (2.2)		

*Statistically significant at alpha = 0.05.

Abbreviations: TTS, time-to-surgery; IPTW, inverse probability-treatment weighting; BMI, body mass index; IQR, interquartile range; SMD, standardized mean difference; CCI, Charlson comorbidity index; ASA, American Society of Anesthesiologists; WBC, white blood cell; Hb, hemoglobin; PLT, platelet; ALP, alkaline phosphatase; PADUA, Preoperative Aspects and Dimensions Used for an Anatomical; UISS, University of California Los Angeles Integrated Staging System.
